# Allopurinol-Induced Toxic Epidermal Necrolysis

**DOI:** 10.7759/cureus.52222

**Published:** 2024-01-13

**Authors:** Sofia Perdigão, Ana Sofia Alves, Mariana Nunes, Cristiana Sousa, Nelson Barros

**Affiliations:** 1 Internal Medicine, Centro Hospitalar de Trás-os-Montes e Alto Douro (Hospital Center of Trás-os-Montes and Alto Douro), Chaves, PRT; 2 Intensive Care Unit, Centro Hospitalar de Trás-os-Montes e Alto Douro (Hospital Center of Trás-os-Montes and Alto Douro), Vila Real, PRT

**Keywords:** toxicity, allopurinol hypersensitivity, stevens-johnson syndrome (sjs), toxic epidermal necrolysis (ten), allopurinol

## Abstract

Toxic epidermal necrolysis (TEN) is a rare and life-threatening cutaneous disease, frequently triggered by drugs. Allopurinol is one of the most frequent drugs associated with TEN, which implies detachment of a significant amount of the body surface area (BSA) and has a high morbidity and mortality associated with it. We present the case of a 68-year-old female with a recent diagnosis of hyperuricemia who started treatment with allopurinol. A week later, she presented to the emergency department with an extensive maculopapular exanthema with blisters and skin detachment. After the exclusion of other etiologies, the diagnosis of allopurinol-induced TEN was made, with 35% of BSA involvement. Due to the severity of the clinical condition, she was admitted to intensive care and treated with corticoids that had no response. So, she was started on immunoglobulins and transferred to a burn unit. She developed sepsis with multiorgan failure and required supportive treatment. She was discharged after a month, and physical rehabilitation was needed. This clinical case highlights the severity of allopurinol hypersensitivity that may happen and the importance of an accurate diagnosis and treatment for this rare disease.

## Introduction

Toxic epidermal necrolysis (TEN) is also called Lyell’s syndrome due to Lyell’s description in 1956 [1]. It is a rare and life-threatening disease that is immunologically mediated and frequently triggered by drugs. It’s characterized by the rapid development of blistering exanthema and purpuric macules, accompanied by extensive necrosis, mucosal involvement, and detachment of the epidermis in variable grades [1]. Stevens-Johnson syndrome (SJS) is also a condition that shares the same pathophysiology as TEN and is classified according to body surface area (BSA) involvement. Toxic epidermal necrolysis involves the detachment of more than 30% of BSA, while SJS comprises less than 10% of BSA, and there is a SJS/TEN overlap that involves between 10% and 30% of BSA [1-3]. Toxic epidermal necrolysis can occur at any age, is more common in women, and has a high morbidity and mortality rate.

Allopurinol is the principal drug used to treat hyperuricemia; it's cost-efficient. The principal mechanism of action is the inhibition of xanthine oxidase, the enzyme involved in the oxidation of hypoxanthine and xanthine, reactions that result in the production of uric acid. It is predominantly metabolized in the liver and then excreted by the kidneys [4-6]. However, allopurinol can cause adverse effects, from a mild form of allopurinol hypersensitivity with maculopapular eruptions to rare, severe cutaneous reactions like TEN.

The risk factors for developing allopurinol-induced TEN are the recent introduction of allopurinol, the presence of the HLA-B*58:01 allele, a higher beginning dose, renal impairment, and the concomitant use of diuretics [4]. Here, we present the clinical case of a woman who started treatment with allopurinol for hyperuricemia and developed a severe, life-threatening cutaneous condition of allopurinol-induced TEN.

## Case presentation

**Figure 1:**
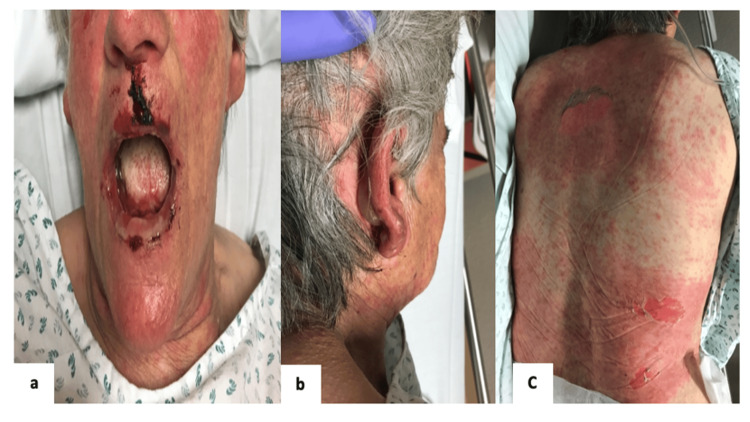
Multiple bullous lesions involving the face and oral mucosa (a), ears (b), and trunk (c)

**Figure 2:**
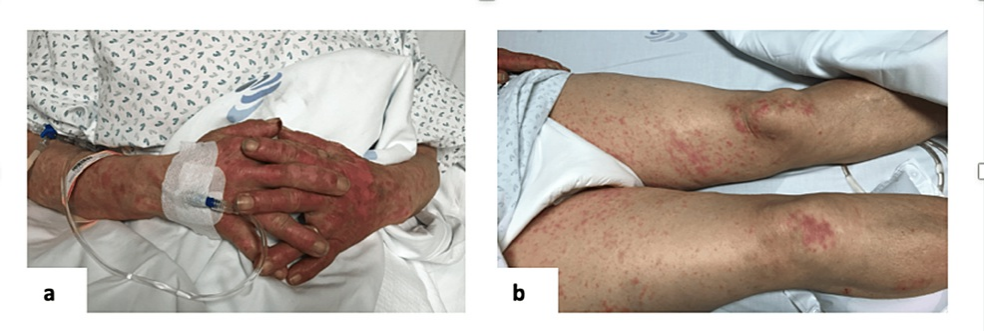
Bullous lesions on limbs

The patient was a 68-year-old female with a past medical history of hypertension, atrial fibrillation under anticoagulation, heart failure, epilepsy, and a recent diagnosis of hyperuricemia. She didn’t have a past history of hypersensitivity or allergy to any drugs. She presented to the emergency department with an extensive and painful exanthema, accompanied by fever, malaise, myalgia, and bilateral purulent conjunctivitis, three days ago. Eight days prior to presentation, she had started treatment with allopurinol 100 mg for hyperuricemia. On physical examination, she presented with facial erythema, bilateral purulent conjunctivitis, involvement of oral mucosa with hemorrhagic erosions, ear bullae, and diffuse maculopapular exanthema with bullae on the thorax (Figure 1) as well as hands and limbs (Figure 2). 

**Figure 3:**
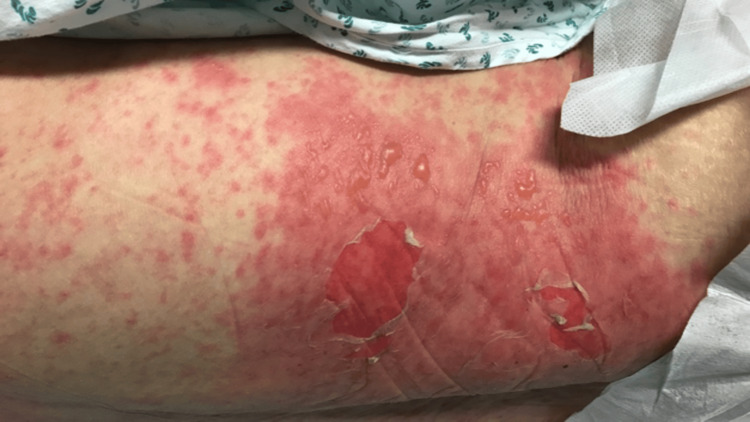
Nikolsky sign positive on the abdomen

The skin was tender to touch with detachment, and a Nikolsky sign on the abdomen and hands was present (Figure 3). She also had a fever (40ºC), tachycardia, and tachypnea.

On laboratory tests, she had thrombocytopenia and an acute kidney injury. The glucose, electrolytes, hepatic function, and coagulation were normal (Table 1). The thorax radiography didn’t show any abnormal findings. Blood and urine cultures were ordered, and the patient was started on empiric antibiotics with amoxicillin clavulanate 1200 mg every eight hours and fluid therapy. An evaluation by ophthalmology was performed, and corneal involvement was excluded. Also, an otorhinolaryngology evaluation was performed, and hemorrhagic erosions on the oral mucosa, palate, and arytenoid glands were confirmed. During her stay in the ward, other etiologies were excluded, such as infections (bacterial/viral/fungi/parasites), neoplasia, and autoimmune disease.

**Table 1:** Analytical results aPTT: Partially activated thromboplastin time; AST: Aspartate aminotransferase; ALT: Alanine aminotransferase; CRP: C-reactive protein; PT: Prothrombin time

Parameters		Value	Reference value
Hemogram	Hemoglobin	12.6 g/dl	12-15 g/dl
	Leucocytes	11.7 (10³/uL)	3.5-10.5 (10³/uL)
	Platelets	99 (10³/uL)	150-400 (10³/uL)
Biochemistry	Glucose	169 mg/dl	70-100 mg/dl
	Creatinine	2.60 mg/dl	0.6-1.2 mg/dl
	Urea	64 mg/dl	10-40 mg/dl
	AST	28 U/L	0-35 U/L
	ALT	32 U/L	0-35 U/L
	CRP	7.8 mg/dl	<0.5 mg/dl
Coagulation	aPTT	24.9 sec	24-35 sec
	PT	14.6 sec	14.8- 16sec

The extent of mucocutaneous lesions was evaluated to be 35% of BSA. During the process of taking a detailed history, the patient mentioned the recent start of allopurinol, and so the diagnosis of allopurinol-induced TEN was made. The prognostic score of TEN (SCORTEN) [3] was 3 points, with a mortality risk of 35.8%. The patient started treatment with corticoid prednisolone 1 mg/kg/day, silver sulfadiazine, and chlorhexidine and was admitted to intensive care.

The evolution wasn’t favorable; she developed urosepsis from Klebsiella pneumoniae. Immunoglobulins for three days were added to the previous treatment (2 gr/kg/day), and she was transferred to a burn intensive care unit due to the worsening of mucocutaneous lesions. The patient stayed for a month with septic shock and multiple organ dysfunction at the time of admission to the unit and required invasive ventilator support and broad-spectrum antibiotics. Eventually, the patient did have a favorable evolution and began re-epithelialization. After discharge, she required physical rehabilitation.

## Discussion

Allopurinol is an effective therapy for hyperuricemia; it's cost-efficient and therefore has good adherence [4]. The side effects are variable, ranging from mild to severe symptoms; in rare cases, about 2% of patients develop severe cutaneous reactions, like TEN. It’s important to monitor and evaluate patients who start treatment with this drug. Also, it’s important to know the risk factors for the development of allopurinol-induced TEN, which include factors related to the time of starting treatment, genetics, drug concentration, the starting dose, and renal impairment or concomitant use of diuretics.

Our patient had started treatment with 100 mg of allopurinol, which is a small dose, and she had normal kidney function and wasn’t under therapy with diuretics. The genetic test for the HLA-B*58:01 allele (a risk factor for TEN) wasn’t performed in this case. The expected time for the onset of an allopurinol-induced severe cutaneous reaction is three weeks, but it can also happen immediately or until 180 days later. According to the literature, it commonly occurs between four days and four weeks after exposure to the drug [5]. Our patient developed symptoms eight days after beginning allopurinol therapy.

Toxic epidermal necrolysis is a rare, life-threatening disease that requires an accurate diagnosis, which should include an efficacy assessment of the disease’s extension and aggressive treatment to achieve a good outcome [7-9]. When a patient presents to the emergency department with a fever (40ºC) and malaise, a painful rash on the face and chest, and mucosal involvement in the eyes and mouth, as in our case (Figures 1-2), these are typical cutaneous manifestations of TEN. An extensive investigation should be made, including laboratory, imaging, immunologic, and genetic tests. A skin biopsy can be made to achieve the correct diagnosis and exclude other diseases.

The 68-year-old patient in our case report initially presented with severe and painful cutaneous lesions, bilateral conjunctivitis, involvement of the oral mucosa with hemorrhagic erosion, blisters on the ear, and a diffuse maculopapular exanthema with blisters on the thorax that spread to the arms, hands, and inferior limbs. The skin was detached at the touch, and the Nikolsky sign [10] was present on the trunk, abdomen, and hands (Figure 3). The extent of cutaneous lesions was 35% of BSA.

The SCORTEN is a validated prognostic score used to predict the probability of hospital mortality in patients with TEN. The score is based on seven independent prognostic factors (Table 2), and one point is attributed to each parameter. Positive higher scores predict higher mortality. The patient had three points per the SCORTEN calculation, predicting a 35.8% mortality rate [3,9,11].

**Table 2:** SCORTEN score SCORTEN: Score of TEN, TEN: Toxic epidermal necrolysis, BSA: Body surface area

Factor	Points
Age > 40 years	1
Heart rate > 120/min	1
Underlying malignancy	1
Skin detachment >10% of BSA on day one	1
Serum urea > 28mg/dL (10 mmol/L)	1
Serum bicarbonate < 20 mEq/L (20 mmol/L)	1
Serum glucose > 250 mg/dL (14 mmol/L)	1

Regarding the treatment for SJS/TEN, the mainstays are the withdrawal of the etiologic drug, supportive therapy with fluids, control of electrolytes, and prevention or control of infection. It’s important to decide where to allocate the patient's best care. According to the UK guidelines of the British Association of Plastic, Reconstructive, and Aesthetic Surgeons published in 2016 [9], patients with involvement of >10% BSA should be admitted to an intensive care unit. For this reason, and due to the extensive skin detachment of our patient, she was admitted to the intensive care unit.

There are few studies about the efficacy of adjuvant therapies in the treatment of this condition. Our patient was on corticoids, but the initial response wasn’t satisfactory, and she was impaired with cutaneous lesions. Immunoglobulins were added to her treatment, resulting in improvement and a good outcome. Reviewing the literature, based on a meta-analysis performed by Tsai et al. [12], the combination of corticoids and immunoglobulins showed statistically significant improvements in outcomes [2,12].

## Conclusions

Allopurinol is a common drug used to treat hyperuricemia. It usually has innocent side effects but, in rare cases, can cause TEN. Toxic epidermal necrolysis is a rare, life-threatening cutaneous disease with systemic inflammation that requires prompt diagnosis and treatment. It’s important before starting treatment with allopurinol to pay attention to risk factors for developing TEN, and in a high-risk population, the screening of the HLA-B*58:01 allele should be performed. In allopurinol-induced TEN, the prognosis and severity of the disease depend upon the amount of skin detachment; the prognostic score, SCORTEN, should be performed on day 1. Considering the severity of the disease, the patient should be admitted to an intensive care unit due to the high risk of complications and mortality.

It’s fundamental to have more studies on treatment approaches, including pharmacological and non-pharmacological therapies to treat TEN. Especially since a lack of evidence can compromise the outcome. With the documentation of this case, the authors intend to alert physicians to both the severity and rare adverse reactions of allopurinol that result in TEN, as well as the complications associated with this diagnosis, such as septic shock.
